# Births in two different delivery units in the same clinic – A prospective study of healthy primiparous women

**DOI:** 10.1186/1471-2393-9-25

**Published:** 2009-06-22

**Authors:** Britt Ingeborg Eide, Anne Britt Vika Nilsen, Svein Rasmussen

**Affiliations:** 1 Department of Obstetrics and Gynaecology, Haukeland University Hospital, Bergen, Norway; 2 Bergen University College, Faculty of Health and Social Sciences, Department of Postgraduate Studies, Bergen, Norway; 3 Institute of Clinical Medicine, University of Bergen, Bergen, Norway

## Abstract

**Background:**

Earlier studies indicate that midwife-led birth settings are associated with modest benefits, including reduced medical interventions and increased maternal satisfaction. The generalizability of these studies to birth settings with low intervention rates, like those generally found in Norway, is not obvious. The aim of the present study was to compare intervention rates associated with labour in low-risk women who begin their labour in a midwife-led unit and a conventional care unit.

**Methods:**

Eligible participants were low-risk primiparas who met the criteria for delivery in the midwife-led ward regardless of which cohort they were allocated to. The two wards are localised at the same floor. Women in both cohorts received the same standardized public antenatal care by general medical practitioners and midwifes who were not involved in the delivery. After admission of a woman to the midwife-led ward, the next woman who met the inclusion criteria, but preferred delivery at the conventional delivery ward, was allocated to the conventional delivery ward cohort. Among the 252 women in the midwife-led ward cohort, 74 (29%) women were transferred to the conventional delivery ward during labour.

**Results:**

Emergency caesarean and instrumental delivery rates in women who were admitted to the midwife-led and conventional birth wards were statistically non-different, but more women admitted to the conventional birth ward had episiotomy. More women in the conventional delivery ward received epidural analgesia, pudental nerve block and nitrous oxide, while more women in the midwife-led ward received opiates and non-pharmacological pain relief.

**Conclusion:**

We did not find evidence that starting delivery in the midwife-led setting offers the advantage of lower operative delivery rates. However, epidural analgesia, pudental nerve block and episiotomies were less often while non-pharmacological pain relief was often used in the midwife-led ward.

## Background

In many places world-wide all births are concentrated to larger maternity clinics, regardless of whether the woman is seen as a healthy, low-risk woman, or whether there are underlying illness or other risk factors existent. During recent decades, particularly in parts of the world with thriving private practice, obstetricians have increasingly taken over responsibility for normal birth [1,2]. Concomitantly, routine use of intervention such as episiotomy, electronic foetal monitoring and pain control by systemic agents, that are not evidence based [3] and ignore the World Health Organization's (WHO) guidelines on the care of women giving birth [1], has increased. As labour intervention and fear of litigation has become more widespread, so too has operative delivery like caesarean section [4]. The WHO has estimated that almost 15% of all women develop complications serious enough to require expedite and skilled intervention if they are to survive without lifelong disabilities [5]. Selection of obstetric care based on risk assessment of the woman admitted to the labour ward has been recommended [1,6-8]. Since the 1970s, during a time of increase in the routine use of technology during labour, alternative birth settings for low-risk women have been established in or near conventional labour wards for the care of pregnant women who prefer and require little or no medical intervention during labour [9].

A Cochrane review of all six randomized or quasi-randomized controlled trials compared intervention rates in conventional institutional birth settings and low risk midwife-led units [10]. Midwife-led settings were associated with modestly reduced medical interventions and increased maternal satisfaction. The generalizability of these studies to birth settings with low intervention rates like those generally found in Norway [11] is not obvious. In such countries one might expect smaller differences in intervention rates between conventional institutional birth settings and low risk midwife-led units.

The aim of the present study was to compare intervention rates associated with labour in low-risk women who begin their labour in a midwife-led unit and a conventional care unit.

## Methods

This was a prospective, non-randomized observational study approved by the regional ethical committee and the Norwegian Data Inspectorate.

### Organisation of Delivery Wards

In the Department of Obstetrics and Gynaecology, Haukeland University Hospital, a total of about 5000 women are giving birth annually. In 1995 the maternity section was reorganised through the establishment of a midwife-led ward (MLW) for women with low risk for complications with the capacity of 1500 deliveries per year. Thus, 3500 deliveries take place in the conventional delivery ward (CDW) per year. Main characteristics of the two wards are presented in Table 1. The MLW and the CDW are localised at the same floor. The MLW, which is a combined delivery and post-partum care unit, is managed by midwives who emphasize the normality of the birth process with minimal intervention in labour. Partners and other support persons are encouraged to take an active role in physical and emotional support. Measures to help manage pain like movement, massage, use of shower/bathtube and acupuncture are available as is pharmacological pain relief with opiates. The post-partum period is spent in the family room and the women are attended by the same personal from admittance to discharge. About 50% of the women spend the post-partum period at a patient hotel by own preference or because of shortage of room. Women who are cared for at the MLW must be healthy with uncomplicated pregnancies without significant congenital malformations or foetal/placental disease and must have regularly attended antenatal care. Women with rupture of the chorioamnionic membranes more than 24 hours, thrombophilia, haemophilia, or drug or alcohol abuse are not admitted to the MLW. Epidural analgesia is not available at the MLW. If the woman needs epidural analgesia during labour she is transferred to the CDW, but still belongs to the MLW cohort. The MLW and CDW share the same legally responsible obstetricians. However, at the MLW obstetricians are consulted only in event of complications during labour and are otherwise not involved in daily management. The two wards have different midwifery staff. During the study period, there was no rotation of midwifes between the two units.

**Table 1:** Characteristics of Care During Labour in Conventional Delivery- and Midwife-led Wards

	CDW	MLW
	
Patients	Low and high risk	Low risk
Numbers of deliveries/year	3500	1500
Antenatal care	By GPs or midwifes at standard antenatal clinic	By GPs or midwifes at standard antenatal clinic
Environment	Conventional hospital	Home-like
Intra- and postpartum care	Intrapartum only	Intra- and postpartum
Philosophy of care	No explicit written philosophy	Supporting natural childbirth, written philosophy of care
Staff	Midwifes	Midwifes
	Obstetricians	Obstetricians consulted in events of complications
Induction of labour	Yes	No
Augmentation of labour	Yes	No
Pharmacological pain relief	Opiates	Opiates
	Pudendal analgesia	Pudendal analgesia
	Nitrous oxide	
	Epidural analgesia	
Non-pharmacological pain relief	Shower	Shower
	Bath	Bath
	Movement/massage	Movement/massage
	Acupuncture	Acupuncture
Transfer	No	In case of medical complication or request for epidural analgesia in the first stage of labour

CDW: Conventional delivery ward

MLW: midwife-led ward

Pregnant women are selected for delivery at the MLW at different times during pregnancy, mostly according to their own preference if they meet the conditions for admittance to the ward. However, the final selection occurs at admittance for labour at the reception ward by a midwife. If the woman requests or needs epidural analgesia at arrival to the reception ward, she is admitted to the CDW.

In the second stage of labour, women were not transferred from the MLW to the CDW unless there was need of emergency caesarean section. The tertiary care CDW with about 3500 labours per year is equipped and staffed for dealing with severe medical situations during labour. Also in the CDW midwifes attend low risk births and obstetricians are usually not present, but are called in case of complications. As in the MLW, pain relief like movement, massage, shower, bathtube, acupuncture, pudental nerve block and opiates were available as were nitrous oxide, epidural analgesia (Table 1), but because of older facilities, shower and bath were to a less extent available than at the low-risk ward.

Women in both cohorts received the same standardized public antenatal care by general medical practitioners and midwifes who were not involved in the delivery.

### Inclusion Criteria

To ensure comparability the study was limited to primiparous women. Eligible participants were low-risk primiparous women who met the criteria for delivery in the MLW regardless of whether they were allocated to the MLW- or CDW cohort, and were admitted to labour between 36 and 42 weeks of gestation.

### Exclusion Criteria

Expressed desire of epidural analgesia at admission to the hospital before admission to the labour ward.

### Participants

**Figure 1:**
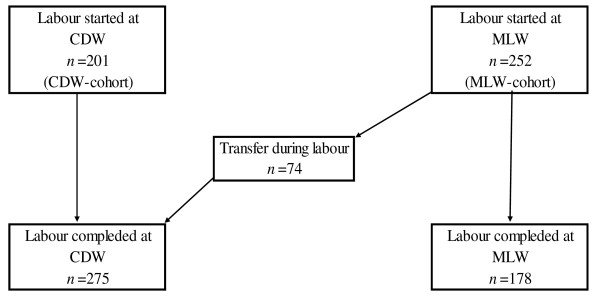
**Study population**. CDW: Conventional delivery ward. MLW: Midwife-led ward

Allocating the participants, we used as strict as possible alternation between the cohorts. After admission of a woman to the MLW, the next woman who met the inclusion criteria, but preferred delivery at the CDW, was allocated to the CDW cohort. During the study, there were no changes in the inclusion criteria. This observational study did not affect the choice of place of birth. 252 and 201 women were included in the MLW and CDW cohorts, respectively (Figure 1). Among the 252 women in the MLW cohort, 74 (29%) women were transferred to the CDW during labour. Thus, 178 and 275 (201 + 74) women completed their labour at the MLW and CDW, respectively.

### Data collection

The following background data were collected from the pregnancy and hospital records and entered into a modified form which was used in a previous study [8]: maternal age, marital status, educational level and cigarette smoking, reason for admittance, cervix dilatation (cm) and information on the actual pregnancy and birth (estimated day of confinement by the last menstrual period and by ultrasound dating, duration of first and second stage of labour, birth position, analgesia during labour, intervention such as epidural analgesia, other methods of pain relief, operative delivery, maternal and foetal complications and Apgar scores).

For each cohort identical forms were completed, except for reason for transfer to the CDW from the MLW during labour. From admission to discharge the forms were completed by one of two project workers who were midwives. Before the data were computerized they were routinely checked for systematic errors. For both cohorts, data collection started November 3, 2001. Data collection was completed May 31, 2002 for the MLW and October 1, 2002 for the CDW cohort. For the CDW cohort, the period of data collection was extended because many otherwise eligible women expressed need of epidural analgesia at admission and were thus not included.

### Statistical analysis

The statistical analyses were carried out with SPSS (Statistical Package for the Social Sciences, SPSS Inc, Chicago, IL, USA). Differences between birth invention rates and outcomes in the cohorts were assessed by Chi square or Fisher's exact test where appropriate and logistic regression. Unadjusted and adjusted odds ratios (ORs) for maternal age, smoking habits, educational level and marital status with 95% confidence intervals (CI) were calculated. Time from regular contractions to delivery and duration of the second stage of birth in the cohorts were compared after ln-transformation to normality.

Pre-study assessment of sample size, given an event rate of 10% in the CDW cohort, indicated a study size of 200 subjects in each cohort.

## Results

Maternal characteristics in both cohorts are shown in Table 2. Women in the CDW cohort were more often smokers. More women in the MLW cohort worked throughout pregnancy. The educational level in the MLW cohort was higher and more women were recorded as cohabitants. No significant difference in the distribution of maternal age categories between the two cohorts was found.

**Table 2:** Maternal Characteristics in the CDW- and MLW-Cohorts

		Cohort				
			
Characteristic		CDW *n *= 201		MLW *n *= 252		*P*-value
			
		n	%	N	%	
Maternal age (years)	<29	12	6.0	10	4.0	0.7
	20–24	56	27.9	62	24.6	
	25–29	77	38.3	109	43.3	
	30–34	44	21.9	57	22.6	
	>34	12	6.0	14	5.6	
						
Marital status	Cohabiting	168	83.6	232	92.1	0.014
	Single motherhood	29	14.4	19	7.5	
	Other or unknown	4	2.0	1	0.4	
						
Education	Elementary	24	11.9	15	6.0	<0.0001
	Upper secondary	91	45.3	82	32.5	
	University	85	42.3	134	53.2	
						
Worked during Pregnancy	Yes	154	76.6	232	92.1	<0.0001
	No	45	22.4	19	7.5	
						
Smoker at 1.st prenatal visit	Non-smoker	143	71.1	208	82.5	0.003
	1–10 cigarettes/day	56	27.9	39	15.5	
	> 10 cigarettes/day	0	0.0	4	1.6	
	Unknown	2	1.0	1	0.4	

CDW: Conventional delivery ward

MLW: midwife-led ward

Place of birth was planned before admittance in 162 (81%) and 203 (81%) women in the CDW and MLW cohorts, respectively (data not presented). Among reasons for the choice of giving birth at the CDW were availability of pain relief other than in the MLW (*n *= 103; 51%) and belief that the ward was safer for mother and infant (*n *= 42; 21%). Among reasons for the choice of the MLW were facilities like bathroom near the delivery room and a generally positive impression of the ward after having been shown around the ward or it had been recommended by women with experience from the wards (*n *= 114; 45%). Other emphasised the possibility of 'natural' birth (*n *= 37; 15%) and that they preferred pain relief without analgesia (*n *= 17; 7%).

The rates of operative delivery in the cohorts were statistically equal (Table 3); the caesarean section rate was 6–7%, the rate of delivery by forceps 4–5% and by vacuum extraction 7–8%. As expected, among those who were transferred intrapartum from the MLW to the CDW (*n *= 74) the use of operative delivery was generally higher (caesarean section (*n *= 16; 22%), forceps (*n *= 10; 14%) or vacuum (*n *= 5; 7%)) (Data not presented). There were three main reasons for transfer to the CDW during labour: Need of epidural analgesia according to the woman's preference or on medical indication (*n *= 31; 42%), need of cardiotocography (*n *= 22; 30%) and protracted labour (*n *= 10; 14%). After adjustment, the difference between the cohorts in the proportions of women who had an episiotomy was marginally significant, 36% in the CDW and 29% in the MLW cohort.

**Table 3:** Interventions in the CDW- and MLW-Cohorts

	CDW		MLW						
	*n *= 201	%	*n *= 252	%	OR	95% CI	OR*	95% CI	
	
Mode of delivery									
Spontaneous vaginal	161	80.1	205	81.3	0.9	0.6–1.5	0.9	0.6–1.5	
Forceps	8	4.0	12	4.8	0.8	0.3–2.1	0.8	0.3–2.0	
Vacuum extraction	16	8.0	17	6.7	1.2	0.6–2.4	1.6	0.7–3.5	
Emergency caesarean section	14	7.0	16	6.3	1.1	0.5–2.3	1.0	0.5–2.2	
Pharmacological pain relief									
Epidural analgesia	126	62.7	61	24.2	5.3	3.5–7.9	4.9	3.2–7.4	**
Opiates	6	3.0	42	16.7	0.2	0.1–0.4	0.1	0.05–0.3	**
Nitrous oxide	49	24.4	10	4.0	7.8	3.8–15.9	7.7	3.7–16.1	**
Pudental nerve block	22	10.9	10	4.0	3.0	1.4–6.4	3.5	1.6–7.8	**
Non-pharmacological pain relief									
Acupuncture	17	8.5	93	36.9	0.2	0.1–0.3	0.2	0.1–0.3	**
Sterile water injections	9	4.5	25	9.9	0.4	0.2–0.9	0.4	0.2–0.99	**
Bathing	85	42.3	164	65.1	0.4	0.3–0.6	0.4	0.3–0.6	**
Episiotomy	73	36.3	72	28.6	1.4	0.97–2.1	1.6	1.05–2.4	**

CDW: Conventional delivery ward

MLW: midwife-led ward

*Adjusted for: maternal age, smoking, education and marital status

OR: odds ratio

CI: confidence interval

** significant after adjusting

In the CDW cohort the most used birth position was half sitting (*n *= 141, 70%) and side position (*n *= 31; 15%). In the MLW cohort positions were more various and the most used were kneeling position (*n *= 35; 14%), birthing stool (*n *= 31; 12%), half sitting (*n *= 22, 9%), side position (*n *= 20; 8%) and use of 'sacco-sack' (*n *= 17; 7%) (data not presented).

Use of analgesia in the two cohorts was different. More women in the CDW cohort received epidural analgesia, nitrous oxide or pudental nerve block, while other methods of pain relief were more often used in the MLW cohort (Table 3).

In the two cohorts, time from regular contractions to delivery and duration of the second stage of labour, rates of excessive post-partum bleeding (=1000 ml), Apgar scores <7 5 minutes postpartum and transfer to the neonatal intensive care unit were statistically non-different. After adjustment, the rates of perineal tears grades III-IV in the two cohorts (*n *= 22, 11% and *n *= 34, 14% in the CDW and MLW, respectively) and intact perineum (without tears grade I-IV and episiotomy) were also statistically non-different (data not presented).

## Discussion

The main findings of the present study was that operative delivery rates associated with labour in women who were admitted to midwife-led and conventional birth wards were statistically non-different. Furthermore, epidural analgesia, pudental nerve block and episiotomies were less often and non-pharmacological pain relief more often used in the MLW cohort.

The possibility for pregnant women to choose between the two delivery wards was established and well known in the community before the present study began. Therefore, randomized allocation to the cohorts would have been difficult. However, next to a randomized design, a controlled design like the present, with as strict as possible alternation between women allocated to the cohorts, would likely be the most effective. A strength of the study are we were able to adjust for possible confounders. However, the effects of adjusting were modest.

An important issue is comparability between the two cohorts. This study was prospective and adhered to the entry criteria which did not change during the study, however, without randomized allocation to the cohorts. The hospital serves the total birth population in the city and therefore our results can likely be applied to low risk populations Norway in general and other affluent countries. There were no losses to follow up. The vicinity of the CDW to the MLW has likely reduced other influence than that of type of care and thus made the cohorts more comparable. However, the choice of giving birth at the MLW may to some extent have been influenced by the vicinity. Therefore, it cannot be ruled out that the lack of difference in operative delivery rates were caused by selection bias. Some women may have chosen, or been advised during antenatal care, to give birth at the MLW because of the safety of having the CDW near by, but would otherwise have chosen admittance to the CDW. This would render the two cohorts more similar than randomized cohorts and possible differences in operative delivery rates would be diluted. However, differences between randomized cohorts in intervention rates would have to be small to be diluted to non-significance. Thus, our results were most likely not caused by selection bias.

The results in the present study are consistent with studies included [12-17] in a Cochrane review from 2005 which assessed the effects of care in midwife-led and conventional ward settings [10]. Compared with conventional ward settings in the review, midwife-led settings were associated with modest benefits, such as reduced use of epidural analgesia and episiotomy, but non-different instrumental delivery- or caesarean section rates. Another recent Cochrane review [18] compared midwife-led models of both ante- and intrapartum care with other models of care. The review reported that midwife-led care was associated with benefits such as reduced use of regional analgesia, fewer episiotomies and instrumental births and concluded that all women should be offered midwife-led models of care and women should be encouraged to ask for this option. However, because the review included both ante- and intrapartum care models, the results are not obviously comparable with the results in the present study. Another important issue is comparison of maternal satisfaction which, however, was outside the scope of the study.

In both cohorts, the risk of operative delivery, and particularly of caesarean section, was low. So too is the risk of operative delivery in general in the country [11]. Because of the generally low rate of operative delivery, it might be argued that it would be unreasonable to expect an even lower operative delivery rate among those women who prefer to deliver in an alternative birth environment, with facilities for expedite intervention available when necessary.

Women who started delivery at the MLW less often had epidural analgesia and pudental nerve block and more often had non-pharmacological pain relief. This may to some extent be explained by the woman's preference for analgesia and type of delivery ward. However, our results indicate a successful adherence to restrictive use of epidural analgesia and pudental block in women who were allocated to the MLW-cohort. The low rates of epidural analgesia and pudental nerve block may result from increased support during labour.

The high occurrence of severe perineal lacerations in both cohorts is an issue of concern. The lack of difference between rates of severe lacerations in the cohorts was unexpected, because epidural analgesia was used much more often in the CDW cohort and different birth positions were used. Earlier studies indicate that use of epidural analgesia is associated with an increased risk of severe perineal laceration [19], however not consistently [20].

The high transfer rate to the CDW (29%) has also been found in other studies [10]. The generally high transfer rate from MLWs indicates that women who start labour at a MLW should have easy access to conventional obstetric care. However, it cannot be ruled out that some transfers in the present study occurred because the vicinity of the CDW to the MLW reduced the threshold of transfer.

## Conclusion

We did not find evidence that starting delivery in the midwife-led setting offers the advantage of lower operative delivery rates. However, epidural analgesia, pudental nerve block and episiotomies were less often and non-pharmacological pain relief more used in the MLW cohort.

## Abbreviations

CDW: conventional delivery ward; MLW: midwife-led ward; OR: odds ratio; WHO: World Health Organization.

## Competing interests

The authors declare that they have no competing interests.

## Authors' contributions

BIE and ABVN discussed study design, supervised the collection of data and wrote the report. SR conducted the analyses and edited the report. All authors are guarantors of the paper.

## Pre-publication history

The pre-publication history for this paper can be accessed here:


